# The Effectiveness of Melissa Officinalis L. versus Citalopram on Quality of Life of Menopausal Women with Sleep Disorder: A Randomized Double-Blind Clinical Trial

**DOI:** 10.1055/s-0040-1721857

**Published:** 2021-01-19

**Authors:** Mahboobeh Shirazi, Mohamad Naser Jalalian, Masoumeh Abed, Marjan Ghaemi

**Affiliations:** 1 Maternal, Fetal & Neonatal Research Centre, Tehran University of Medical Sciences, Tehran, Iran; 2 Department of Obstetrics and Gynecology, Yas Hospital, Tehran University of Medical Sciences, Tehran, Iran; 3 Department of Gynecology and Obstetrics, School of Medicine, Alborz University of Medical Sciences, Alborz, Iran; 4 Valie-Asr Reproductive Health Research Center, Tehran University of Medical Sciences, Tehran, Iran

**Keywords:** melissa officinalis l., citalopram, menopause, quality of life, sleep disturbance

## Abstract

**Objective** The present study aimed to assess the effect of *Melissa Officinalis L.* (a combination of lemon balm with fennel fruit extract) compared with citalopram and placebo on the quality of life of postmenopausal women with sleep disturbance.

**Methods** The present study is a randomized, double-blind, placebo clinical trial among 60 postmenopausal women with sleep disturbance who were referred to a university hospital from 2017 to 2019. The participants were randomized to receive *M. Officinalis L.* (500 mg daily), citalopram (30 mg) or placebo once daily for 8 weeks. The Menopause-Specific Quality of Life (MENQOL) questionnaire was self-completed by each participant at baseline and after 8 weeks of the intervention and was compared between groups.

**Results** The mean for all MENQOL domain scores were significantly improved in the *M. Officinalis L.* group compared with citalopram and placebo ( *p* < 0.001). The mean ± standard deviation (SD) after 8 weeks in the *M. Officinalis L.,* citalopram and placebo groups was 2.2 ± 0.84 versus 0.56 ± 0.58 versus 0.36 ± 0.55 in the vasomotor ( *p* < 0.001), 1.02 ± 0.6 versus 0.28 ± 0.2 versus 0.17 ± 0.1 in the psychomotor-social ( *p* < 0.001), 0.76 ± 0.4 versus 0.25 ± 0.1 versus 0.11 ± 0.1 in the physical and 2.3 ± 1.0 versus 0.35 ± 0.5 versus 0.41 ± 0.5 in the sexual domain, respectively.

**Conclusions** The results revealed that *M. Officinalis L.* may be recommended for improving the quality of life of menopausal women with sleep disturbance.

**Trial registration** The present study was registered by the name “Comparison of the efficacy of citalopram and compound of *Asperugo procumbens* and *foeniculum vulgare* in treatment of menopausal disorders” with the code IRCT2013072714174N1 in the Iranian Registry of Clinical Trials (IRCT).

## Introduction

Menopause is a physiologic phase characterized by the permanent cessation of ovulation. 1 During this transitional period, women experience quite a lot of physical, psychological, and social symptoms such as hot flashes, mood variability, and sleep disturbance that may last for years. 2

Sleep duration and quality in menopause may be degraded when compared with premenopausal women, which is presumably due to declines in the sex hormones. 3 The symptoms can range from slight discomfort to intensive and debilitating marks such as insomnia and intermittent sleep. 2 Insomnia appears to be due to night sweats caused by the hormonal changes and lead to an increase in awakening. 4

Sleep disturbance can impact negatively on the quality of life and it seems that vasomotor symptoms are a key component of sleep disruption. 5

It is indicated that hormone replacement therapy can minimize estrogen deficiency, which may lead to an improvement in quality of life in the physical, emotional, and sexual aspects of life. 6 However, due to its known side effects and sometimes the contraindications, using alternative botanical therapies may be beneficial in this group of women. 7

The extract of lemon balm and fennel as *Melissa Officinalis L.* is an Iranian herbal extract with an anxiolytic effect and has a positive impression on sleep disturbance. 8 9 10 Phytochemical investigations revealed that this plant contains volatile compounds, triterpenoids, phenolic acids and flavonoids and effects on mood, cognition and memory. 11 The present study was conducted to determine the effects of this herbal ingredient in improving postmenopausal women's sleep disturbance and quality of life compared with citalopram and placebo.

## Methods

### Trial Registration

The present study was registered under the name “Comparison of the efficacy of citalopram and compound of *Asperugo procumbent* and *foeniculum vulgare* in treatment of menopausal disorders” with the code IRCT2013072714174N1 in the Iranian Registry of Clinical Trials (IRCT).

### Ethics Statements

The protocol of the present study was approved by the ethical committee of the Tehran University of Medical Sciences with the code 89746. The participants subsequently submitted a written consent form to participate in the trial. The present trial was conducted according to the principles of the Helsinki Declaration.

### Study Overview

The present randomized double-blind placebo clinical trial was performed from April 2017 to November 2019 among 60 postmenopausal women who were referred to the Women Hospital (a university hospital affiliated to the Tehran University of Medical Sciences) due to sleeping disturbance. The inclusion criteria were all postmenopausal women (at least 1 year without menstrual cycle) along with confirming tests of menopause (follicle stimulating hormone (FSH) > 40 m IU/mL, and estradiol < 20pg/mL) who were poor sleepers according to the Pittsburgh Sleep Quality Index (global score ≥ 5). The validity and reliability of the Pittsburgh Sleep Quality Index questionnaire in Iran were approved via the study by Nazifi et al. 12 The exclusion criteria were a history of hormone replacement therapy in the 6 weeks before the study, malignancies, thyroid disorders, cardiovascular diseases, lipid abnormalities, diabetes mellitus, and active psychiatric diseases. Indeed, we excluded the women who took the sleeping pills as well as other medications in the last 3 months, which may influence their sleep habits. Within 8 weeks of the assessment, an independent clinician, who was unaware of the allocated groups, performed the assessments. After the enrolment was completed, randomization was done with an allocation sequence generated by block randomization by the trial statistician. The participants were divided into 3 equal groups given a block size of 20. The groups received citalopram (30 mg), an equal combinatorial capsule of lemon balm leaf and fennel fruit named as *M. Officinalis L.* (500 mg) or placebo capsule (containing 500 mg starch) every day for 8 weeks. The pharmaceutical products were packed into similar envelopes and with the same appearance. The capsules were built in the faculty of Tehran Traditional Pharmacy. Participants and providers were blind to the treatment. In the first visit after confirming their sleep disturbance, the participants were evaluated by asking their history, and physical examination was conducted for each subject. Then, they were asked to complete the Menopause-Specific Quality of Life Questionnaire and the demographic checklist including age, the age of menarche, marital age, menopausal age, parity, and body mass index (BMI). Then the providers asked the participants to administer 1 capsule daily for 8 weeks. There were no special requirements or lifestyle recommendations during this course. In the follow-up period, the participants were tracked for the incidence of any side effects. In their last follow-up visit after 8 weeks, they were requested to refill the questionnaire and report any probable side effects of the drugs.

### Assessments Tool

The data were collected using a demographic checklist in the first visit and the Menopause-Specific Quality of Life Questionnaire (MENQOL) in the first and last follow up. The MENQOL was self-administered and was used to investigate the women's quality of life during the last month. This questionnaire contained 29 items in the Likert form, including vasomotor (items 1–3), psychosocial (items 4–10), physical (items 11–26), and sexual (items 27–29) scales. Each question was scored from zero (not annoying at all) to 6 (very annoying). Means were computed by dividing the sum of the domain item by the number of items within the domain. 13 A higher score indicated better quality of life. This questionnaire had good internal consistency in the Persian language in vasomotor, physical and psychosocial domains, but not in the sexual domain. 14 The data were collected and analyzed in a secure framework.

### Statistics

The treatment groups were initially compared on baseline demographic variables with chi-squared tests and independent samples *t* -tests. The one-sample Shapiro-Wilk test was used to test the normal distribution of the data. The Wilcoxon signed-rank test was applied to compare the mean score of the variables before and after the interventions, and the Kruskal-Wallis test was applied for comparing the variables among the three groups. Data were analyzed using Stata software version 12 (Statacorp, College Station, TX, USA). P-values < 0.05 were considered statistically significant.

## Results

In the present study, 60 postmenopausal women were recruited, and no participants left or withdrawed before the termination of the study. There was no significant difference between the mean of the Pittsburgh Sleep Quality Index (PSQI) scores between the 3 groups ( *p* = 0.835). The average age was 51.9 years old (minimum 43, maximum 60 years old). Table 1 compares the mean of age, age of menarche, marital age and menopausal age as well as BMI and parity between the three groups of the study. There was no significant difference between the demographic characteristics of the participants.

**Table 1:** Baseline demographic characteristics by three allocated groups of the participants

	Melissa Officinalis L. **	Citalopram	Placebo	Total	*p-value*
**Age**	51.7 ± 3.3	52.6 ± 3.5	51.4 ± 3.3	51.9 ± 3.4	0567
**Age at menarche**	13.2 ± 2.4	12.8 ± 2.6	12.9 ± 2.5	12.9 ± 2.3	0.890
**Marital age**	21.2 ± 4.2	20.2 ± 3.9	22.5 ± 4.1	21.3 ± 4.1	0.232
**Menopausal age**	50.6 ± 1.4	49.2 ± 1.7	50 ± 1.5	49.9 ± 1.6	0.154
**Bdoy mass index**	25.6 ± 1.1	25.1 ± 1.3	26.2 ± 1.1	25.6 ± 1.2	0.456
**Parity**	4.5 ± 1.4	4.9 ± 1.4	4.4 ± 1.3	4.6 ± 1.3	0.546

** Mean ± standard deviation.

Table 2 indicates the baseline mean values score of MENQOL domains including vasomotor, psychomotor-social, physical and sexual domais, before the intervention. Difficulty in sleeping had a worse score in all groups. Hot flashes with mean ± SD = 7.03 ± 06.08 from the vasomotor domain were the second worst and facial hair from the physical domain had the best score (1.68 ± 1.15). The total score of quality of life by MENQOL between groups was not significantly different before the intervention.

**Table 2:** Mean baseline score of MENQOL domains in participants of the three groups before intervention *

	Melissa Officinalis L.	Citalopram	Placebo	*p-value*
**Vasomotor**	5.83 ± 1.3 **	5.85 ± 1.3	5.95 ± 1.2	0.749
**Psychomotor-Social**	3.84 ± 1.5	3.96 ± 1.6	3.93 ± 1.4	0.661
**Physical**	3.06 ± 1.0	3.52 ± 1.1	3.08 ± 0.9	0.760
**Sexual**	6.56 ± 1.0	5.61 ± 1.6	5.91 ± 1.3	0.812

* Higher index indicates worse quality of life.

** Mean ± standard deviation.

The total score of MENQOL in the 3 groups got better compared with baseline ( *p* ≤ 0.001). Table 3 indicates the mean and percentage of improving the score of MENQOL domains in participants of the three groups after the intervention. As shown in Table 3 , improving the score of quality of life in the *M. Officinalis L.* group was significant ( *p* < 0.001) compared with the other groups, and its highest effect was on the vasomotor domain, with a mean of 2.2 ± 0.8. The citalopram group was also more effective than placebo in improving various domains of quality of life ( *p* < 0.001). In the present study, two participants in the citalopram group reported nausea and one case reported headache, all of which were self-controlled. No adverse effect was reported in the *M. Officinalis L.* and placebo groups.

**Table 3:** Mean ± standard deviation change and percentage change of MENQOL domains after 8 weeks of intervention

	Melissa Officinalis L.		Citalopram		Placebo		*p-value*
	Mean ± SD	Percentage change (%)	Mean ± SD	Percentage change (%)	Mean ± SD	Percentage change (%)	
**Vasomotor**	2.2 ± 0.8	-38.0	0.56 ± 0.5	-11.4	0.36 ± 0.5	-5.7	< 0.001
**Psychomotor-Social**	1.02 ± 0.6	-23.8	0.28 ± 0.2	-6.8	0.17 ± 0.1	-4.1	< 0.001
**Physical**	0.76 ± 0.4	-23.6	0.25 ± 0.1	-7.1	0.11 ± 0.1	-4.0	< 0.001
**Sexual**	2.3 ± 1.0	-34.5	0.35 ± 0.5	-7.6	0.41 ± 0.5	-6.4	< 0.001

Abbreviation: SD, standard deviation.

## Discussion

Sleep disturbance is one of the critical symptoms in menopause that influences quality of life. 15 The main pathology of sleep disruption may be vasomotor symptoms specially in the transitional period. 5 The result of the present study showed that *M. Officinalis L.* is more effective than citalopram and placebo in improving the quality of life of the participants. The vasomotor symptoms as well as the physical, psychomotor-social, and sexual domains of quality of life in the participants improved more in the *M.Officinalis L* . compared with other groups.

The average age of the participants with sleep disturbance was near 52 years old, which may be due to the severity of this problem in the transitional stage and early menopause. 5

It seems that citalopram compared with placebo may decrease insomnia symptoms and improve sleep quality 16 ; but in some reviews, the selective serotonin reuptake inhibitors (SSRIs) or citalopram for the treatment of major depression and anxiety disorders have noted that insomnia is a commonly reported adverse event. 17 18 This conflict in effects of specific SSRIs on self-reported sleep in women may be due to several reasons including differences in the pharmacologic properties of specific drugs, study populations, choice of sleep outcome measures, and reporting of adverse events. 16

The global trend shows that the number of people preferring to use herbal ingredients instead of chemicals is growing. 7 19 *M. Officinalis L.,* as a botanical substance, has anxiolytic effects and improves sleep quality in humans. 20 21 It is also recommended in sleep disturbance in menopause. 22 Indeed, this extract had been tested on animal models to decrease anxiety and as an antioxidant. 20 23 24 The mechanism of action may be due to gamma-aminobutyric acid (GABA) elevation on the brain, 22 25 because it is assumed that GABAergic neurotransmission has been associated with reductions in anxiety, 20 but the extent of its therapeutic effects and amounts of various constituents in the extract is unknown. 18

*M. Officinalis L.* is a safe and well-tolerated ingredient, and no adverse event was reported due to the administration of 500 mg of *M. Officinalis L.* in humans. 26 In our study, no participant reported adverse events or complications during the trial,

The strength of our study may be due to the recommendation of *M. Officinalis L* . to improve the quality of life of menopausal women, which naturally declines in middle age. 27 Besides, this ingredient did not have the same potential side effects as citalopram. 28

It seems that the placebo effect on health-related quality of life is meaningful. 29 In our study, we experienced a placebo effect on quality of life. On the other hand, women who received placebo obtained a better score in MENQOL compared with baseline scores.

Our limitation was the small sample size and short duration of the study. Indeed, based on Ghazanfarpour et al., 30 we did not omit the “Vaginal dryness during intercourse” and “weight gain” items from MENQOL due to missing the consistency of the scores.

## Conclusion

The present study demonstrates that *M. Officinalis L.* may improve the quality of life in menopausal women with sleep disorders. Furthermore, no adverse effects were reported. It is recommended that further research will be undertaken in this field with a larger sample size, longer follow-up, and different racial populations.
